# Case report: Dysphonia associated with high-dose cytarabine therapy

**DOI:** 10.3389/fphar.2025.1518298

**Published:** 2025-01-27

**Authors:** Ling Ma, Niya Huang, Haixi Zhang, Jia Liu, Zhiqing Zhang

**Affiliations:** ^1^ Department of Clinical Pharmacy, The First People’s Hospital of Yunnan Province, The Affiliated Hospital of Kunming University of Science and Technology, Kunming, Yunnan, China; ^2^ Department of Hematology, The First People’s Hospital of Yunnan Province, The Affiliated Hospital of Kunming University of Science and Technology, Kunming, Yunnan, China

**Keywords:** acute myeloid leukemia, dysphonia, high-dose cytarabine, adverse reaction, case report

## Abstract

Cytarabine is widely used in the treatment of hematological malignancies. Its common toxicities include myelosuppression and gastrointestinal disturbances. Additionally, it can cause central nervous system (CNS) symptoms, which include hoarseness, ataxic tremor, ataxic gait, nystagmus, dysmetria, and dysdiadochokinesia. In this article, we present the first case report of dysphonia, absent of CNS symptoms, induced by high-dose cytarabine (HiDAC) in a patient with acute myeloid leukemia. The patient’s voice began to change 3 days following the first cycle of HiDAC chemotherapy, and dysphonia recurred upon the reintroduction of HiDAC. To rule out other potential causes, a thorough examination and detailed medical history review were conducted, excluding factors such as vocal abuse, infection, effects of other medications, and underlying diseases as contributors to the dysphonia. The patient was diagnosed with HiDAC-induced dysphonia. This toxic effect was self-limiting, and the patient recovered in 10–15 days. Chemotherapy-induced dysphonia is a rarely reported and easily overlooked side effect. This adverse reaction is typically temporary and non-life-threatening; however, it substantially diminishes quality of life and may occasionally necessitate the discontinuation or postponement of chemotherapy. Physicians should be aware of this complication when administering chemotherapeutic agents.

## Introduction

Dysphonia in patients with cancer receiving chemotherapy often goes unnoticed in clinical practice since most symptoms are temporary and mild. However, this condition can significantly impair quality of life and, in some cases, may necessitate the discontinuation or postponement of chemotherapy. Cytarabine is believed to induce cytotoxicity through the inhibition of DNA polymerase and has been reported to be efficacious in treating leukemia and other hematologic malignancies with acceptable toxicity (Shimony et al., 2023). Its common toxicities are myelosuppression and gastrointestinal disturbances (nausea and vomiting with occasional diarrhea). It may also cause conjunctivitis, anaphylaxis, pulmonary edema, central nervous system (CNS) symptoms, hepatotoxicity, and drug-related fever (Stentoft, 1990). The following case report describes a patient with acute myeloid leukemia (AML) who developed dysphonia related to high-dose cytarabine therapy, which has not been reported previously.

## Case report

**FIGURE 1:**
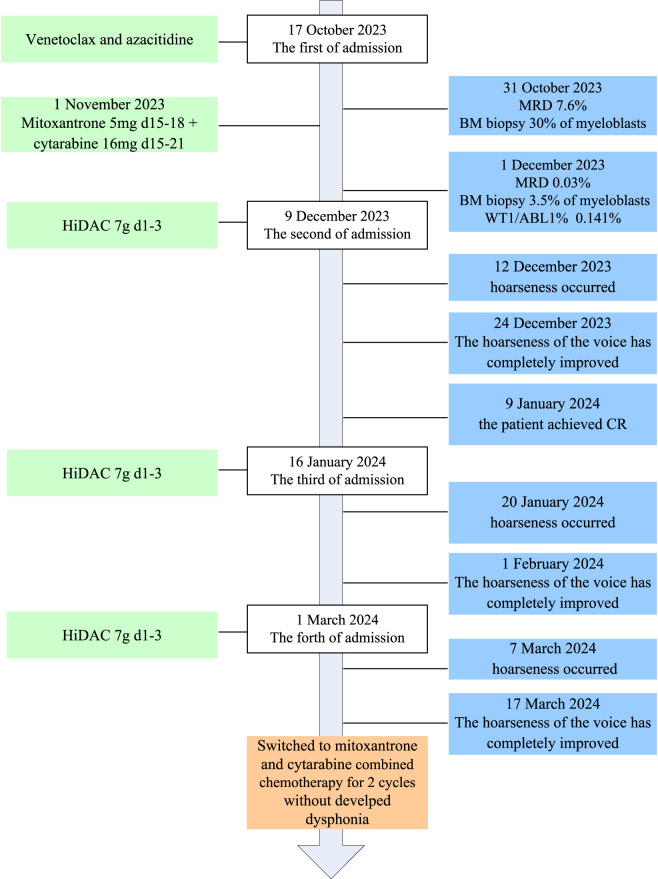
Timeline and treatment process.

A 38-year-old male patient was admitted to our hospital with a 5-month history of leukocytopenia. Laboratory tests revealed the following: hemoglobin 55 g/L, white blood cells 7.1 × 10^9^/L, neutrophilic granulocytes 0.7 × 10^9^/L, and platelets 231 × 10^9^/L. Bone marrow morphology showed that myeloblasts accounted for 31%. Bone marrow biopsy revealed normal bone marrow hyperplasia (60%–70%) with diffuse proliferation of blast cells. Flow cytometry results demonstrated an abnormally high myeloid blast cell population (80.8%), comprising CD45, CD38, CD117, CD34, HLA-DR, CD13, CD33, CD7, CD64, and MPO. The *WT1* gene was positive, followed by RT-PCR; the WT1/ABL1% was 8.052%. The mutations of *FLT3-TKD*, *IKZF1*, *RUNX1*, *JAK1*, *ASXL2*, and *SETD2* were detected. Based on the patient’s medical history and laboratory findings, he was diagnosed with acute myeloid leukemia (high risk) with partial CD7 expression. The course of its treatment is shown in Figure 1. The patient was treated with a combination of venetoclax and azacitidine induction chemotherapy (venetoclax PO once daily 100 mg on day 1, 200 mg on day 2, and 400 mg on days 3–28, and azacitidine 75 mg/m^2^ IV on days 1–7) on 17 October 2023. A bone marrow biopsy performed on day 14 revealed hypocellularity with 30% myeloblasts. Flow cytometry (FCM) indicated a minimal residual disease (MRD) level of 7.6%. On 1 November 2023, the patient was immediately started on the MA regimen (mitoxantrone 5 mg IV on days 15–18 and cytarabine 16 mg IH on days 15–21). One month later, a follow-up bone marrow biopsy showed a blast percentage of 3.5%; with MRD reduced to 0.03% and a WT1/ABL1 ratio of 0.141%, a morphologic leukemia-free state was achieved. Subsequently, intensification therapy with high-dose cytarabine (HiDAC) was begun (cytarabine 4.5 g q12 h IV on days 1–3) on 9 December 2023. The patient’s voice changed 3 days after the HiDAC chemotherapy. Dysphonia symptoms included hoarseness and softness in his voice. The patient denied stridor, cough, dysphagia, or difficulty in breathing during this episode; no tingling or feeling of a foreign body in the throat; and had no history of vocal abuse and symptoms of dysphonia. Risk factors for dysphonia, such as smoking, shouting, coughing, speaking in a loud voice, excessive noise at work, family history of deafness, sleep apnea, gastroesophageal reflux disease, and excessive throat clearing were assessed. A laryngoscopic examination showed normal vocal cords, with normal movements and no lesion or necrosis. In the first week after chemotherapy, type IV bone myelosuppression began to appear without fever or symptoms of infection. The dysphonia was self-limiting, with complete clinical resolution observed after 12 days. One month after chemotherapy, the patient achieved a complete response (CR), and MRD and WT1/ABL1% were negative. Another two cycles of the same therapy were repeated on 16 January and 1 March 2024; however, hoarseness of voice developed synchronously with HiDAC re-treatment. Then, his treatment was switched to MA (mitoxantrone 15 mg IV on days 1–3 and cytarabine 250 mg IV on days 1–7). He tolerated this regimen well, without developing dysphonia.

## Discussion

Chemotherapy-related dysphonia has been reported with anti-angiogenic chemotherapeutic agents, immune checkpoint inhibitors, oxaliplatin, paclitaxel, and vinca alkaloids (Saavedra et al., 2014; Iimura et al., 2021; Berretta et al., 2004; Choi and Robins, 2008; Burns and Shotton, 1998). Dysphonia associated with anti-angiogenic agents and immune checkpoint inhibitors may be caused by hematic crusts, edema, inflammation, neurotoxicity, capillary regression in the laryngeal mucosa, and necrosis in the vocal folds (Saavedra et al., 2014; Melo et al., 2019; Hartl et al., 2012; Roodhart et al., 2008; Carter et al., 2015). Berretta et al. found that dysphonia represents a further aspect of the peripheral neurotoxicity associated with oxaliplatin treatment, which is a self-limiting toxic effect that resolves without treatment (Iimura et al., 2021). Furthermore, in their research, fibrolaryngoscopy was performed in all patients with voice alterations, which was negative for morpho-functional modifications. Neurotoxicity is a common complication of treatment with taxanes and vinca alkaloids; both classes of drugs may cause hoarseness due to vocal fold palsy (Choi and Robins, 2008; Burns and Shotton, 1998).

Upon the initial onset of hoarseness, there was no evidence of an upper respiratory tract infection, and the patient denied any history of vocal abuse. A laryngoscopic examination also revealed no anatomical changes. At this time, the patient was receiving the following medications: cytarabine, ondansetron, tropisetron, dexamethasone, and antacids. Notably, in the previous treatment cycle, the patient had used these same medications without experiencing any hoarseness. It appears that the hoarseness is not associated with the medications, and despite investigation, the underlying cause remains uncertain. During the subsequent administration of HiDAC, the patient experienced a recurrent episode of hoarseness. However, the patient tolerated the MA regimen well, with no development of dysphonia. Based on these observations, we consider the possibility of a dose-dependent adverse reaction associated with cytarabine administration.

Cytarabine-induced dysphonia has been described to occur 3–12 days after the first infusion. According to the Naranjo Adverse Drug Reaction Probability Scale, the score was 7, which indicates that the adverse events were likely adverse reactions to cytarabine (Table 1). It is of interest that the patient developed dysphonia three times with HiDAC treatment and did not develop it with MA which including low-dose cytarabine. This case suggests that cytarabine-induced dysphonia may be dose-dependent. Reasons for such a dosing difference may be related to the neurotoxicity of high-dose cytarabine.

**TABLE 1:** Naranjo Adverse Drug Reaction Probability Scale (Naranjo et al., 1981).

Question	Yes	No	Do not know	Score
1. Are there previous conclusive reports of this reaction?	+1	0	0	**1**
2. Did the adverse event appear after the suspected drug was administered?	+2	−1	0	**2**
3. Did the adverse reaction improve when the drug was discontinued or a specific antagonist was administered?	+1	0	0	**1**
4. Did the adverse event reappear when the drug was re-administered?	+2	−1	0	**2**
5. Are there alternative causes (other than the drug) that could on their own have caused the reaction?	−1	+2	0	**0**
6. Did the reaction reappear when a placebo was given?	−1	+1	0	**0**
7. Was the drug detected in the blood (or other fluids) in concentrations known to be toxic?	+1	0	0	**0**
8. Was the reaction more severe when the dose was increased or less severe when the dose was decreased?	+1	0	0	**1**
9. Did the patient have a similar reaction to the same or similar drugs in a previous exposure?	+1	0	0	**0**
10. Was the adverse event confirmed by any objective evidence?	+1	0	0	**0**
	Total score			**7**

Note: The scores for the Naranjo Adverse Drug Reaction Probability Scale are as follows: Certain >9, Probable 5–8, Possible 1–4, and Unlikely 0. Bold values provided the scores for cytarabine-induced hoarseness.

Cerebral symptoms such as seizures, somnolence, and confusion were noted in the first series of patients treated with HiDAC injections (Rudnick et al., 1979), but these are avoided when the drug is administered by infusion over 1–3 h. Subsequently, a distinct cerebellar toxic syndrome emerged (Barnett et al., 1985; Dworkin et al., 1985; Nand et al., 1986). A cohort study showed that 7 out of 30 patients (23%) studied exhibited CNS toxicity related to the use of HiDAC (Nand et al., 1986). Symptoms began with lethargy and progressed to confusion within hours. Other symptoms and signs of cerebellar dysfunction followed within the next 24 h, including dysarthria, ataxic tremor, ataxic gait, nystagmus, dysmetria, and dysdiadochokinesia. In contrast to these previous reports, our patient experienced only hoarseness without any other symptoms indicative of cerebellar degeneration. Our patient’s laryngoscopic examination did not show any anatomical changes. To the best of our knowledge, this is the first report of a patient developing HiDAC-associated dysphonia without concurrent acute cerebellar dysfunction during AML therapy.

The underlying reasons for this side effect remain unclear. Given the similarity to oxaliplatin-induced peripheral neurotoxicity, we hypothesize that high-dose cytarabine may affect the laryngeal muscles or neuromuscular junction. Although no abnormalities were detected on endoscopy, we consider the possibility of spasmodic dysphonia. This condition, focal dystonia affecting the voice, is marked by intermittent, involuntary spasms of the laryngeal muscles. Although once considered psychogenic due to its association with stress, it is now recognized as having a neuromuscular etiology, the specifics of which are not yet fully understood (House and Fisher, 2017). Further research is warranted to better understand the incidence and mechanisms of dysphonia in patients treated with HiDAC.

## Conclusion

This study highlights the difference from previous reports of HiDAC-related dysphonia. In our case, dysphonia resolved upon discontinuation of the HiDAC therapy. Although dysphonia in cancer patients undergoing chemotherapy is often overlooked in clinical practice due to its typically temporary and mild nature, which is not life-threatening, it can have a significant impact on a patient’s quality of life. In some cases, it may even lead to the discontinuation or postponement of chemotherapy. Therefore, it is crucial for clinicians to recognize and not underestimate this adverse effect, as proper management can improve both patient outcomes and treatment adherence.

## Data Availability

The original contributions presented in the study are included in the article/supplementary material; further inquiries can be directed to the corresponding author.
